# From transfer to receipt: transition in neuromuscular disorders in the era of disease-modifying therapy

**DOI:** 10.1186/s13023-026-04624-8

**Published:** 2026-10-05

**Authors:** Nadia Abdallah, Hans D. Katzberg

**Affiliations:** Division of Neurology, Department of Medicine, University of Toronto, Toronto, ON Canada

**Keywords:** Neuromuscular diseases, Transition to adult care, Rare diseases, Disease-modifying therapy, Registries, Quality indicators, Health policy

**To the Editor**,

We read with great interest the article by Evangelista and colleagues [1], who have performed an important service for the field by quantifying the current state of transition care in neuromuscular disorders across twenty European countries. Their observation that fewer than one in three respondents reported a structured transition protocol, with designated coordinators and formal quality metrics rarer still, provides much-needed evidence of challenges that many programmes have experienced locally for years [1]. It is encouraging that more than half of the respondents were adult neurologists, as durable solutions will require engagement from the receiving side of the transition as much as from the referring side. Our adult neuromuscular programme in Toronto has operated structured referral pathways from paediatric neuromuscular and rehabilitation services since 2010, and our experience closely echoes their findings. We write to offer two observations that may complement the authors’ proposed roadmap.

The first is that these challenges appear to be global rather than European. When we profiled young adults arriving in our own clinics, we documented the same breadth of unmet multidisciplinary need described by the survey respondents, together with recurring difficulties in record transfer and continuity of accountability [2]. A recent mixed-methods study of adults with spinal muscular atrophy, also from Toronto, reported that only one of twenty participants had received a written transition plan and that none had a shared care plan [3]. A parallel survey within the European Reference Network for rare neurological diseases (ERN-RND) found standardised transition procedures in a quarter of participating centres [4]. At the same time, encouraging efforts are underway in several countries: the Essen model in Germany demonstrates the value of a deliberately structured handover [5], the Spanish neurology societies have published national transition recommendations this year [6], and practical guidance has recently appeared in the United States [7]. Nevertheless, a 2026 systematic review in Duchenne muscular dystrophy concluded that most transition initiatives remain project-based rather than embedded in policy [8]. The consistency of these observations across very different health systems suggests that the principal barriers are inherent to the interface between paediatric and adult services, and are unlikely to be overcome by local efforts alone.

The second concerns the changing implications of discontinuity. Much of the transition literature, understandably, has framed interrupted follow-up primarily as a loss of surveillance. In the current therapeutic era, the consequences may be greater. Many young adults now enter adult services while established on disease-modifying treatment for conditions such as spinal muscular atrophy, Duchenne muscular dystrophy, and Pompe disease, in some cases with mandated long-term safety follow-up after gene therapy. The monitoring these treatments require does not end with paediatric care: scheduled assessments must continue across and beyond the transfer, whichever clinic the patient attends, and in some jurisdictions continued reimbursement depends on their documentation, so that a gap in care may also become a gap in treatment eligibility. Whereas a patient lost to follow-up in the past lost continuity of care, a patient lost today may experience interruption of disease-modifying therapy, with the potential for irreversible functional decline. We believe it is reasonable to regard unsuccessful transition as a patient-safety concern, and to manage it accordingly.

This perspective suggests a practical refinement to the roadmap the authors propose. Patient-safety concerns are typically addressed with clear definitions, denominators, and audit, and each could be applied here. We would suggest that transition be considered complete not when a referral is made, but when receipt is confirmed: that is, when a first adult clinic visit has taken place, with medication and monitoring plans reconciled, within a defined interval such as six months from the final paediatric visit, and with the referring centre retaining responsibility until that point. A six-month interval is consistent with existing transition guidance, which recommends confirming completion of transfer within three to six months of the final paediatric visit [9]. Retention could then be verified at twelve and twenty-four months. We intend these as programme-level audit measures rather than as new clinical requirements for individual patients. Importantly, the infrastructure for such measurement already exists. The European Reference Network for rare neuromuscular diseases (ERN EURO-NMD) operates a federated registry built on FAIR (findable, accessible, interoperable, reusable) data principles [10], and a minimal transition dataset embedded within it (Table 1) could provide the standardised outcome measures the authors call for, at little additional cost. Such data would also strengthen the case for dedicated reimbursement of coordination, joint visits, and case management, which the survey identified as a major unmet need [1].

**Table 1:** Proposed minimal transition dataset for rare neuromuscular disease registries

Item	Definition (example target)
Transition initiated	Date of first documented transition discussion (by age 15 to 16 years, in keeping with survey respondents’ preferences [1]
Transfer summary	Standardised, disease-specific summary completed and shared with the adult team (yes/no; date)
Receipt	First completed adult-clinic visit with medication and monitoring plans reconciled (within 6 months of the final paediatric visit)
Therapy continuity	Interruption of disease-modifying therapy or mandated monitoring during the transfer window (yes/no; duration, reason)
Retention	Attending adult specialist care at 12 and 24 months (yes/no)
Unplanned care	Emergency visits or unplanned admissions within 12 months of transfer (count)
Experience	Patient- or family-reported transition experience at the first adult visit (validated measure of choice)

Should ERN EURO-NMD proceed to define a common transition dataset, harmonisation with programmes outside Europe, including our own, would be feasible and worthwhile, as it would allow transition outcomes in rare neuromuscular disease to be compared systematically across health systems. Given the rarity of these disorders, no single centre or health system will accumulate sufficient experience in isolation. Evangelista and colleagues have provided the field with a timely evidence base, and we support their call for a coordinated roadmap in which transition care is defined, measured, and adequately resourced.

## Data Availability

No datasets were generated or analysed during the current study.

## Abbreviations

ERN EURO-NMD: European reference network for rare neuromuscular diseases
ERN-RND: European reference network for rare neurological diseases
FAIR: Findable, accessible, interoperable, reusable
